# Adiponectin Ameliorates Experimental Periodontitis in Diet-Induced Obesity Mice

**DOI:** 10.1371/journal.pone.0097824

**Published:** 2014-05-16

**Authors:** Lan Zhang, Shu Meng, Qisheng Tu, Liming Yu, Yin Tang, Michel M. Dard, Sung-Hoon Kim, Paloma Valverde, Xuedong Zhou, Jake Chen

**Affiliations:** 1 Division of Oral Biology, Tufts University School of Dental Medicine, Boston, Massachusetts, United States of America; 2 Key Laboratory of Oral Diseases, West China Hospital of Stomatology, Sichuan University, Chengdu, Sichuan, China; 3 Periodontology and Implant Dentistry, New York University College of Dentistry, New York, New York, United States of America; 4 Cancer Preventive Material Development Research Center (CPMDRC) and Institute, College of Oriental Medicine, Kyung Hee University, Dongdaemun-gu, Seoul, Korea; 5 Department of Sciences, Wentworth Institute of Technology, Boston, Massachusetts, United States of America; 6 Department of Anatomy and Cell Biology, Tufts University School of Medicine, Sackler School of Graduate Biomedical Sciences, Boston, Massachusetts, United States of America; University of Toronto, Canada

## Abstract

Adiponectin is an adipokine that sensitizes the body to insulin. Low levels of adiponectin have been reported in obesity, diabetes and periodontitis. In this study we established experimental periodontitis in male adiponectin knockout and diet-induced obesity mice, a model of obesity and type 2 diabetes, and aimed at evaluating the therapeutic potential of adiponectin. We found that systemic adiponectin infusion reduced alveolar bone loss, osteoclast activity and infiltration of inflammatory cells in both periodontitis mouse models. Furthermore, adiponectin treatment decreased the levels of pro-inflammatory cytokines in white adipose tissue of diet-induced obesity mice with experimental periodontitis. Our *in vitro* studies also revealed that forkhead box O1, a key transcriptional regulator of energy metabolism, played an important role in the direct signaling of adiponectin in osteoclasts. Thus, adiponectin increased forkhead box O1 mRNA expression and its nuclear protein level in osteoclast-precursor cells undergoing differentiation. Inhibition of c-Jun N-terminal kinase signaling decreased nuclear protein levels of forkhead box O1. Furthermore, over-expression of forkhead box O1 inhibited osteoclastogenesis and led to decreased nuclear levels of nuclear factor of activated T cells c1. Taken together, this study suggests that systemic adiponectin application may constitute a potential intervention therapy to ameliorate type 2 diabetes-associated periodontitis. It also proposes that adiponectin inhibition of osteoclastogenesis involves forkhead box O1.

## Introduction

Periodontitis is an inflammatory disease that involves progressive loss of alveolar bone around the teeth and can result in tooth loss. It is twice as prevalent in diabetics as in non-diabetics, and has been rated as the sixth complication of diabetes [1]. Pathologically and clinically, type 2 diabetes (T2D)-associated periodontitis is more severe than in non-diabetics. Excess white adipose tissue (WAT) in obese is characterized by increased macrophage infiltration and production of proinflammatory cytokines including tumor necrosis factor-α (TNF-α) and interleukine-6 (IL-6) that mediate local and systemic effects on inducing insulin resistance [2]. Indeed, this systemic inflammation and insulin resistance in T2D contributes to the pathogenesis of periodontitis [1], [3]. Hence developing an effective therapeutic treatment for T2D-associated periodontitis that can both inhibit bone resorption and decrease inflammation is critically important.

Adipose tissue plays an important role in energy homeostasis by secreting a number of adipokines, among which adiponectin (APN) [4] has been shown to exhibit insulin-sensitizing effects [4]–[6], and potent anti-inflammatory properties [7]. Circulating levels of APN are reduced in obesity, T2D or periodontitis [8]–[11], whereas improvement in hyperglycemia of T2D upon treatment with thiazolidinediones [12], [13] or periodontal therapeutic intervention that decreased inflammation, significantly led to increased serum APN levels [14]. Low circulating levels of this adipokine may therefore be related to insulin resistance and poor periodontal status.

Bone metabolism involves the concerted actions of bone resorbing cells called osteoclasts [15] and bone producing cells called osteoblasts [16]. While lack of APN in APN knockout (APN^−/−^) mice did not result in an obvious bone phenotype change [17], [18]; when bone was explanted into these mice, noticeable effects of APN on bone metabolism were revealed. Thus, lack of APN led to significant growth retardation of bone explants and increasing osteoclastogenesis [19]. APN has been shown to indirectly stimulate osteoclast differentiation via receptor activator of nuclear factor kB ligand (RANKL) and osteoprotegerin (OPG) expression by osteoblasts [20] and to inhibit osteoclast activity and bone resorption by suppressing RANKL-induced Akt signaling in osteoclasts [19], [21]. Furthermore, APN can decrease bone mass by inhibiting osteoblast differentiation and promoting their apoptosis via inducing phosphorylation of Akt which downregulated forkhead box O1 (FoxO1) [22]. In addition APN was shown to increase bone mass by decreasing sympathetic tone [22]. Taken together, the peripheral and central effects of APN on bone metabolism require further investigation.

In this study we established experimental periodontitis in mice to evaluate whether systemic APN infusion could ameliorate periodontal destruction in APN^−/−^ and diet-induced-obesity (DIO) mice, a model of obesity and T2D. Furthermore, we performed *in vitro* studies with osteoclast precursor cells to delineate the molecular mechanisms implicated in APN signaling under osteoclastogenic conditions.

## Materials and Methods

### Ethics Statement

The animal protocols used in this study were approved by the Institutional Animal Care and Use Committee at Tufts University/Tufts Medical Center (Approved Protocol #B2011-49). All mice were kept in a controlled temperature-and controlled room under a 12 h light, 12 h dark cycle.

### Purification of Recombinant APN Protein and Periodontal Pathological Bacteria

pEt15b bacterial expression vector encoding the C-terminal part of human APN (amino acids 106–244) was used to purify globular APN as a His-tagged protein in BL21(D3) bacterial cells as described previously [23]. *Porphyromonas gingivalis* (*P. gingivalis*, ATCC) was cultured and maintained in supplemented tryptic soy broth (ATCC) in an anaerobic chamber with 85% N_2_, 10% H_2_, and 5% CO_2_ at 37°C. 5–0 silk sutures were presoaked in the broth containing (10^8^/ml) *P. gingivalis* for 2 days prior to periodontitis induction.

### Mice, Experimental Periodontitis Induction, and Systemic APN Infusion

Male APN^−/−^ (Jax #008195), DIO (Stock #380050), and wild-type (WT, Jax #000664) mice were purchased from the Jackson Laboratory (Bar Harbor, ME, USA). DIO mice were fed with 60% high fat diet.

APN^−/−^ mice were randomly divided into 3 groups (n = 5/group): experimental periodontitis (PD), experimental periodontitis with systemic APN treatment (PD+APN), and untreated mice (control). WT mice were divided into 2 groups (n = 5/group): experimental periodontitis (PD), and untreated mice (control). DIO mice were divided into 2 groups (n = 5/group): experimental periodontitis (PD), and experimental periodontitis with systemic APN treatment (PD+APN).

To induce experimental periodontitis [24], [25], mice were anesthetized by an IP injection of ketamine (80 mg/kg) and xylazine (10 mg/kg). A 5–0 silk suture presoaked in the bacterial broth for two days was then wrapped around the right and left second maxillary molars and knotted mesio-buccally. Ligatures were changed every other day to maintain sufficient microbial burden.

As the APN half-life is only 2.5 h, APN was administered by systemic infusion in order to guarantee a constant blood concentration. An Alzet micro-osmotic pump (model 1004, Durect Corporation) was subcutaneously inserted in the back of each mouse following periodontitis induction. Sham surgery for osmotic pump insertion was performed in the animal models used in this study (data not shown). The APN concentration in the pump was 1 mg/ml and the pump rate was set at 0.11 µl/h, hence the pump delivered approximately 2.5 µg of recombinant APN per day to each mouse. Mice were euthanized 10 days after periodontitis induction.

### Cell Culture and Transfection Experiments

RAW264.7 (ATCC) cells were cultured in RPMI 1640 with 10% fetal bovine serum (FBS, Life Technologies). Cells were serum-starved overnight and treated with receptor activator of NF-κB ligand (RANKL, PeproTech), *E.coli* lipopolysaccharide (LPS, Sigma-Aldrich), or the c-Jun N-terminal kinase (JNK) inhibitor SP600125 (Tocris Bioscience). Transfection of plasmids was performed using Lipofectamine 2000 (Life Technologies) following the manufacturer’s recommendations. The pGL3-CtpsK-luciferase reporter vector was constructed in our previous study [19], which contained a 4.0-kb mouse cathepsin K promoter. Plasmid encoding FoxO1 (Flag-FoxO1) was purchased from Addgene (Cambridge, MA). pCMV5 which contained the vector backbone was used as a control plasmid in transfection experiments.

### Quantitative Real-Time PCR (qRT-PCR) for mRNA Analyses

Total RNA from RAW264.7 cultures were prepared with an RNeasy Mini Kit (Qiagen) and reverse-transcribed with M-MLV Reverse Transcriptase (Affymetrix) according to the manufacturer’s instructions. qRT-PCR assays were performed with USBVeriQuestFastSYBRGreenqPCR Master Mix with Fluorescein (Affymetrix) using a Bio-Rad iQ5 thermal cycler. The mRNA expression levels of target genes were calculated with the comparative cycle threshold method using GAPDH as a control.

WAT was removed from male DIO mice. Total RNA was prepared from tissues with TRIzol reagent (Life Technologies) according to the manufacturer’s instructions. Reverse transcription and qRT-PCR assays were performed as mentioned above to detect the expression of TNF-α, interleukin-1 (IL-1), and IL-6 in WAT. Primers used for amplification are listed in Table 1.

**Table 1:** Primers used in qRT-PCR analyses.

Gene		Primer Sequence
GAPDH	Forward	5′-AGG TCG GTG TGA ACG GAT TTG-3′
	Reverse	5′-TGT AGA CCA TGT AGT TGA GGT CA-3′
TNF-α	Forward	5′-CAT CTT CTC AAA ATT CGA GTG ACA A-3′
	Reverse	5′-TGG GAG TAG ACA AGG TAC AAC CC-3′
IL-6	Forward	5′-GAG GAT ACC ACT CCC AAC AGA CC-3′
	Reverse	5′-AAG TGC ATC ATC GTT GTT CAT ACA-3′
IL-1	Forward	5′-CCA TGG CAC ATT CTG TTC AAA-3′
	Reverse	5′-GCC CAT CAG AGG CAA GGA-3′
Cathepsin K	Forward	5′-GAA GAA GAC TCA CCA GAA GCA G-3′
	Reverse	5′-TCC AGG TTA TGG GCA GAG ATT-3′
FoxO1	Forward	5′-CTC CCG GTA CTT CTC TGC TG-3′
	Reverse	5′-GTG GTC GAG TTG GAC TGG TT-3′

GADPH, Glyceraldehyde-3-phosphate dehydrogenase; TNF-α, tumor necrosis factor-alpha; IL-6, interleukin-6; FoxO1, forkhead box O1.

### Western Blot Analyses

Whole protein lysates were prepared with RIPA lysis buffer (Santa Cruz Biotechnology, Inc.) according to the manufacturer’s instructions. Nuclear proteins were purified using a nuclear extraction kit (EMD Millipore). SDS-PAGE electrophoresis and Western blots were performed using Novex 4–20% Tris-Glycine gels (Life Technologies) and 0.45 µm polyvinylidene fluoride membranes (Millipore). Antibodies for nuclear factor of activated T cells c1 (NFATc1, 1∶1000) and lamin B1 (1∶1000) were purchased from Santa Cruz Biotechnologies. FoxO1 (1∶1000), p-JNK (1∶1000), and JNK (1∶1000) were purchased from Cell Signaling Technology. The secondary antibodies were horseradish peroxidase-linked goat-anti-rabbit IgG (Santa Cruz Biotechnology, Inc.). Blots were visualized using SuperSignal West Dura Extended Duration Substrate (Thermo Fisher Scientific).

### Luciferase Assay

Co-transfections of Flag-FoxO1 (or pCMV5 control plasmid) with pGL3-CtpsK-luciferase reporter vector were performed in RAW264.7 cells and 50 ng/ml RANKL was used to induce osteoclastogenesis for 5 days. Then luciferase assay was performed using a Lumat LB9501 luminometer (Berthold Technologies) as described previously [26].

### *In Vitro* Osteoclastogenesis and Tartrate-Resistant Acid Phosphatase (TRAP) Staining

Primary cultures of mouse osteoclast precursor cells in the form of bone marrow-derived monocytes/macrophages (BMM) were obtained from 6–8 week-old male WT mouse femurs and tibias as described previously [27]. Briefly, femurs and tibias were flushed with a 25-gauge needle and cultured in α-MEM supplemented with 10 ng/ml monocyte colony-stimulating factor (M-CSF) overnight. The non-adherent cells were collected and layered on Histopaque gradient (Sigma). Cells at the gradient interface were seeded to 96 well plates. Transfection of FoxO1 or pCMV5 control plasmid was performed. Then osteoclastogenesis was induced with 10 ng/ml M-CSF and 50 ng/ml RANKL. The medium was changed every 3 days. After 7 days of induction, cells were fixed and stained for TRAP activity using the K-ASSAY TRACP staining kit (Kamiya Biomedical Company). The osteoclasts were identified as red-stained cells with three or more nuclei. The number of osteoclasts was manually counted in four separate fields at a magnification of 200×. Data were reported as the mean number of osteoclasts in one separate field.

### Alveolar Bone Loss Analysis

After euthanasia, the palatal bone samples were dissected and defleshed after 15 minutes in boiling water, immersed overnight in 3% hydrogen peroxide, and stained with 1% methylene blue. The buccal and palatal faces of the molars were photographed at 30× magnification using a dissecting microscope with the occlusal face of the molars positioned perpendicular to the base. The distance from the cementoenamel junction to the alveolar crest was measured at six sites of secondary molar: mesio-buccal, mid-buccal, disto-buccal, disto-palatal, mid-palatal and mesio-palatal using Image-Pro Plus software [24], [25].

### Histology and TRAP Staining of Bone Specimens

Palatal bone samples were fixed in 4% paraformaldehyde and decalcified in 10% EDTA. Tissue sections were stained with hematoxylin and eosin (H&E), and interdental areas between the first and second molars were examined. The total number of inflammatory cells, mainly polymorphonuclear leukocytes, was manually counted according to its morphology, from 4 separate fields at 400× magnification on H&E-stained sections. Data were reported as the numbers of inflammatory cells per square millimeter. TRAP staining was performed using Acid Phosphatase, Leukocyte (TRAP) kit (Sigma-Aldrich) according to the manufacturer’s instructions. The osteoclasts were identified as red-stained cells with three or more nuclei. The number of osteoclasts was counted from images captured at 200× magnification. Data were presented as the number of osteoclasts per bone surface.

### Statistical Analysis

Data are presented as the average ± standard deviations (SD) of 3 or more experiments. Statistical significance was analyzed by one-way ANOVA followed by LSD post hoc test or independent sample t test; and data were considered significant at *P*<0.05.

## Results

### APN Inhibits Alveolar Bone Resorption and Infiltration of Inflammatory Cells in APN^−/−^ Mice Induced with Experimental Periodontitis

**Figure 1:**
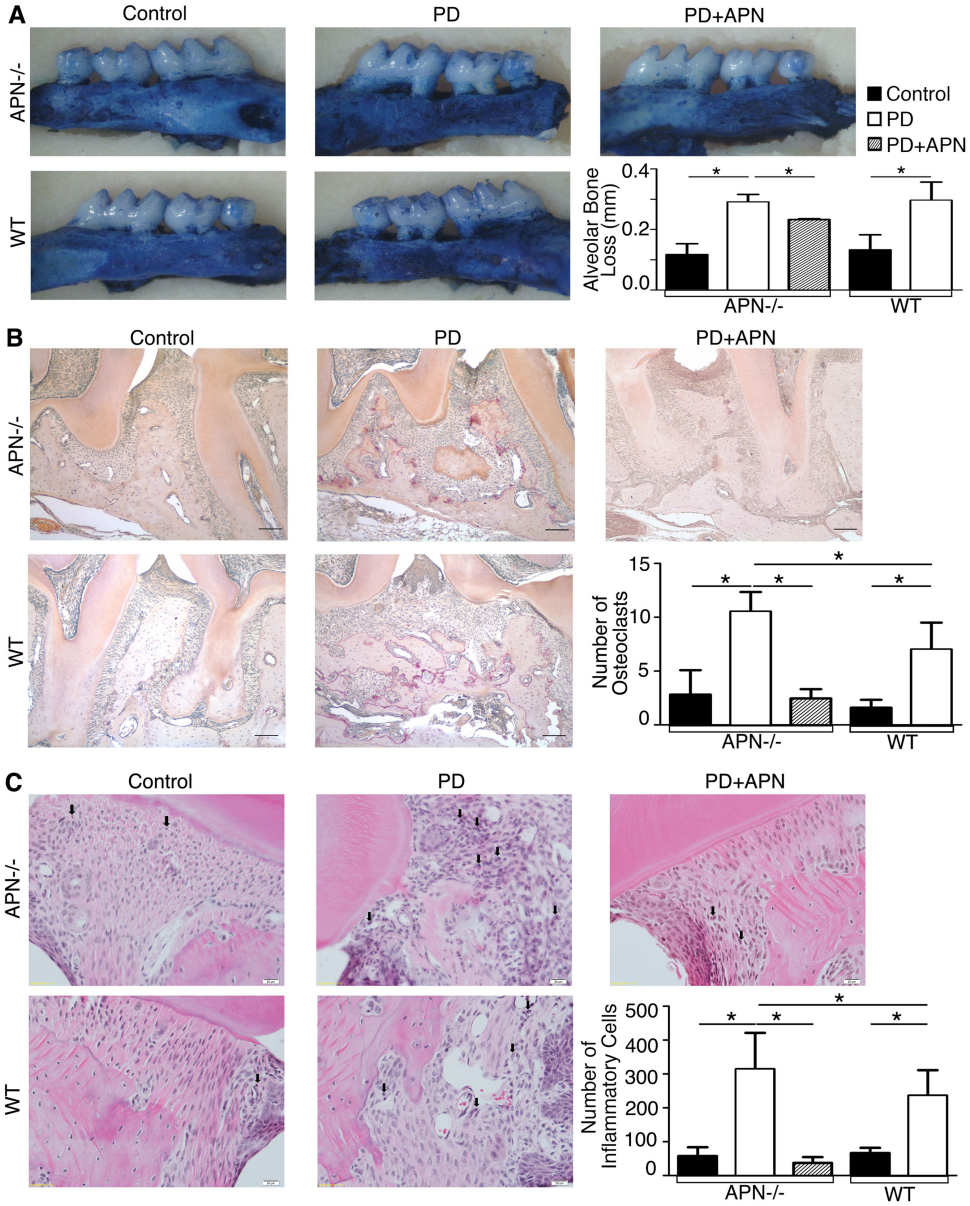
APN Inhibits Bone Resorption and Inflammation in APN^−/−^ Mice Induced With Experimental Periodontitis. (A) Alveolar bone loss was determined in palatal bone samples stained with 1% methylene blue and photographed at 30× magnification using a dissecting microscope with the occlusal face of the molars perpendicular to the base. The distance between the cementoenamel junction and the alveolar crest was measured at 6 sites in APN^−/−^, APN^−/−^+PD, APN^−/−^+PD+APN, as well as WT and WT+PD mice (magnification, ×30). (B) TRAP staining determined the number of osteoclasts (magnification, ×200; scale bars, 500 µm). (C) H&E staining determined the number of inflammatory cells of palatal bone samples in APN^−/−^, APN^−/−^+PD, APN^−/−^+PD+APN, as well as WT and WT+PD mice (magnification, ×400; scale bars, 20 µm; black arrows = inflammatory cells). Data are shown as mean ± SD (n = 5). *P<0.05.

Several lines of evidence have revealed that APN can act directly and indirectly in bone metabolism through complex regulatory mechanisms. To investigate the plausible therapeutic effects of APN on alveolar bone loss, we established experimental periodontitis in APN^−/−^ and WT mice. Our results showed that alveolar bone loss was significantly increased upon induction of experimental periodontitis in male WT and APN^−/−^ mice (Figure 1A, *P*<0.05), indicating that periodontitis was successfully established. Although there was no significant difference in alveolar bone loss between WT and APN^−/−^ mice induced with periodontitis, TRAP-stained palatal bone samples from APN^−/−^ mice with periodontitis exhibited a higher number of osteoclasts than in those derived from WT mice induced with experimental periodontitis (Figure 1B, *P*<0.05). Furthermore, periodontitis induction led to higher infiltration of inflammatory cells in APN^−/−^ mice than in WT mice (Figure 1C, *P*<0.05), suggesting that APN deficiency made mice susceptible to periodontal destruction. Then we administered recombinant APN in APN^−/−^ mice and found that systemic APN infusion significantly decreased alveolar bone loss associated with experimental periodontitis in APN^−/−^ mice (Figure 1A, *P* *<*0.05). More importantly, systemic APN treatment reduced the number of osteoclasts (Figure 1B, *P*<0.05) and infiltration of inflammatory cells (Figure 1C, *P*<0.05) in APN^−/−^ mice induced with periodontitis.

### APN Inhibits Alveolar Bone Resorption, Infiltration of Inflammatory Cells and Expression of Proinflammatory Cytokines by WAT in DIO Mice Induced with Experimental Periodontitis

**Figure 2:**
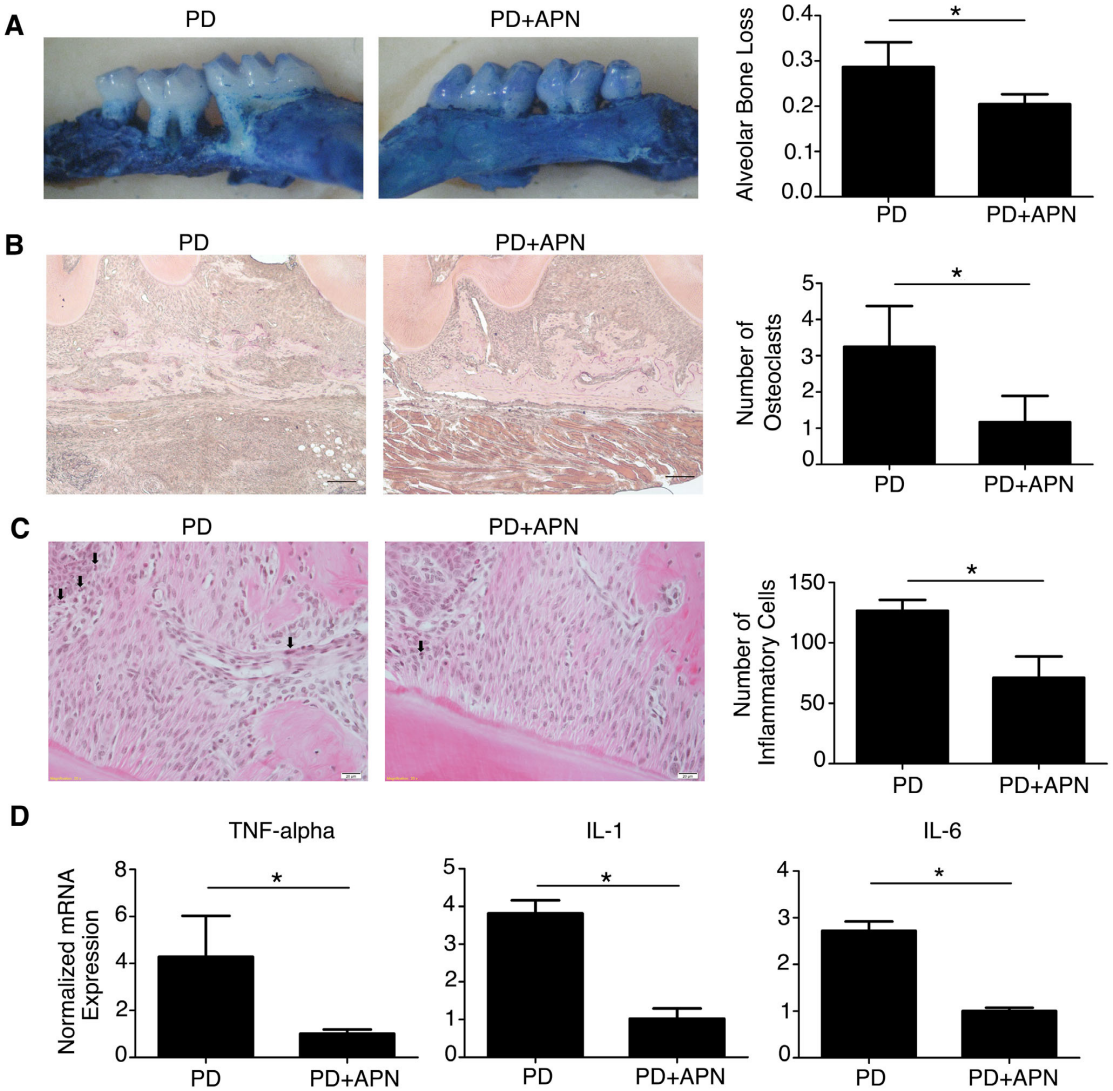
APN Inhibits Bone Resorption and Inflammation in DIO Mice Induced With Experimental Periodontitis. (A) Alveolar bone loss was determined in palatal bone samples stained with 1% methylene blue and photographed at 30× magnification using a dissecting microscope with the occlusal face of the molars perpendicular to the base. The distance between the cementoenamel junction and the alveolar crest was measured at 6 sites in DIO+PD and DIO+PD+APN mice (magnification, ×30). (B) TRAP staining determined the number of osteoclasts (magnification, ×200; scale bars, 500 µm). (C) H&E staining determined the number of inflammatory cells of palatal bone samples in DIO+PD and DIO+PD+APN mice (magnification ×400; scale bars, 20 µm; black arrows = inflammatory cells). (D) qRT-PCR of TNF-α, IL-1, and IL-6 mRNA levels in WAT from DIO+PD and DIO+PD+APN mice, normalized to GAPDH. Data are shown as mean ± SD (n = 5). *P<0.05.

T2D patients are more prone to developing severe periodontitis [1], [3]. To mimic the pathological characteristics of periodontitis in T2D patients, we induced experimental periodontitis in DIO mice. DIO mice are a widely studied model of T2D. These mice develop obesity with elevated blood glucose and impaired glucose tolerance when fed a high-fat diet. We then evaluated whether systemic APN infusion could mediate potential therapeutic effects on alveolar bone loss, osteoclast number and infiltration of inflammatory cells in palatal bones of DIO mice induced with periodontitis. Our results revealed that systemic APN infusion significantly downregulated alveolar bone loss (Figure 2A, *P*<0.05), osteoclast number (Figure 2B, *P*<0.05), and the number of inflammatory cells (Figure 2C, *P*<0.05) in DIO palatal bones. We also found that systemic infusion of APN in the DIO-animal model diminished hyperglycemia (data not shown), which was in agreement with the insulin sensitizing properties of APN.

Excess WAT in obesity has been linked to insulin resistance partly through production of proinflammatory cytokines by infiltrated macrophages [2], [28]. The insulin resistance and chronic general inflammation in T2D patients could ultimately contribute to periodontitis that is more severe and refractory in T2D patients than in patients without diabetes [1], [3]. We then evaluated whether systemic APN infusion affected the mRNA expression of proinflammatory cytokines by WAT isolated from DIO mice with periodontitis. Our results indicated that recombinant APN treatment decreased mRNA expression of the proinflammatory cytokines including TNF-α, IL-1, and IL-6 in WAT isolated from DIO mice induced with periodontitis (Figure 2D, *P*<0.05).

### APN Promotes FoxO1 Expression and Nuclear Activation in RANKL-Treated RAW264.7 Cells

**Figure 3:**
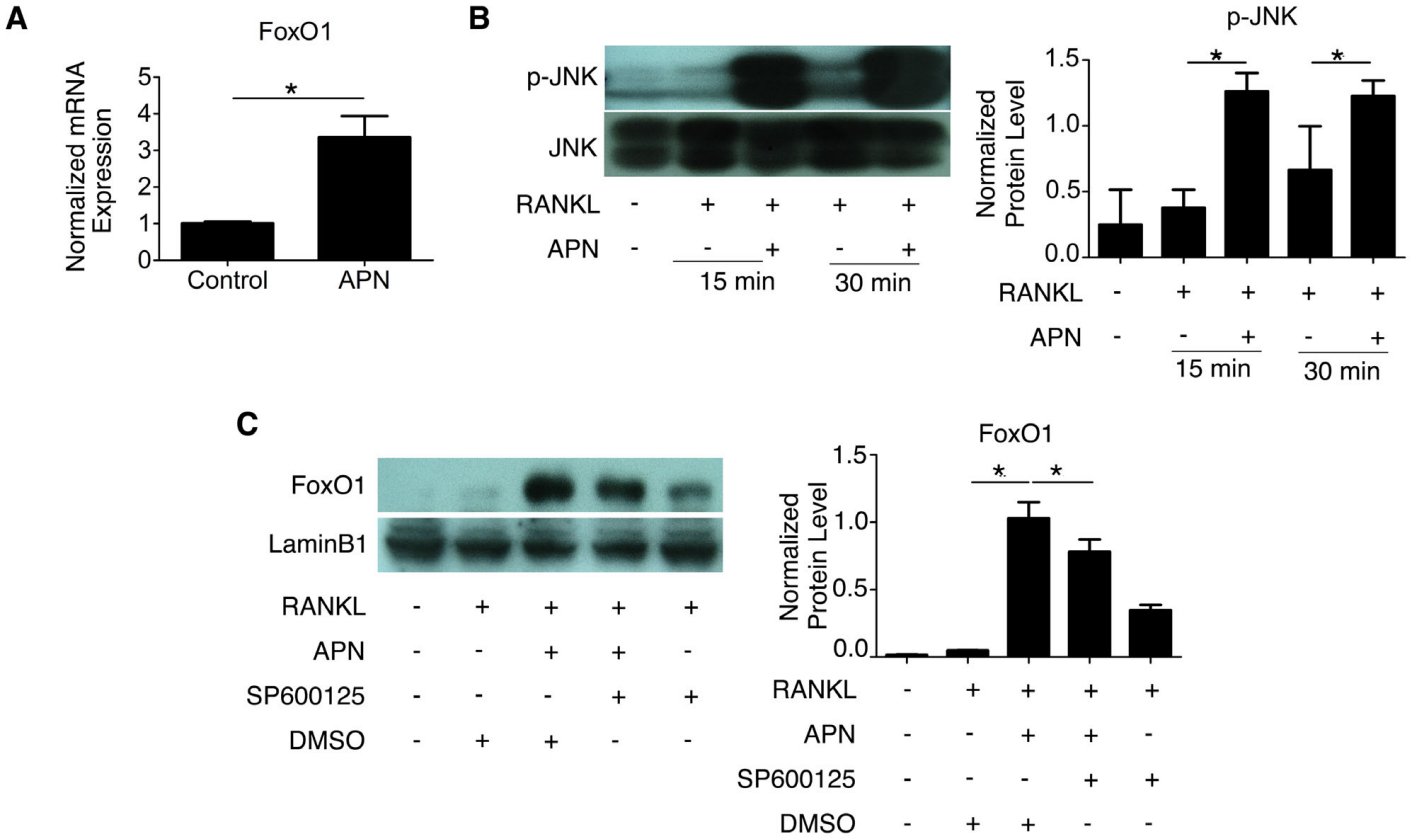
APN Induces FoxO1 and JNK Phosphorylation in RANKL-Treated RAW264.7 Cells. (A) qRT-PCR of FoxO1 mRNA levels in RAW264.7 cells treated with 50 ng/ml RANKL in the absence (control) and presence of 0.5 µg/ml APN treatment for 24 hours. The mRNA level was normalized with those of GAPDH. (B) Western blot for phosphorylated JNK in RAW264.7 cells treated with 50 ng/ml RANKL in the absence or presence of 0.5 µg/ml APN for 15 or 30 minutes. JNK was detected as the loading control. Data are shown as mean ± SD of three independent experiments. *P<0.05. (C) Western blot of FoxO1 nuclear protein extracted from RAW264.7 cells that were treated with 50 ng/ml RANKL in the presence and absence of 0.5 µg/ml APN, with or without prior treatment with the JNK inhibitor SP600125 for 2 hours. DMSO was used to dissolve SP600125. Nuclear Lamin B1 was used as the loading control. Data are shown as mean ± SD of three independent experiments. *P<0.05.

FoxO1 is a forkhead transcription factor that acts as the master regulator of energy metabolism [28], and it is highly expressed in insulin-responsive tissues including bone. On one hand, FoxO1 controls glucose metabolism through osteoblasts by regulating the activity of osteocalcin, and on the other it serves as a target of insulin signaling in osteoblasts. Recently APN was also reported to decrease bone mass by decreasing the nuclear levels of FoxO1 in osteoblasts [22]. We then investigated whether APN could also inhibit osteoclastogenesis by directly signaling through FoxO1 in osteoclasts. To that end, we first analyzed the FoxO1 mRNA expression in RAW264.7 cells undergoing RANKL-induced osteoclastogenesis in the presence and absence of APN. We found that the normalized FoxO1 mRNA expression was significantly upregulated in cells treated with APN (Figure 3A, *P*<0.05). As JNK has been implicated in activating FoxO1 by increasing the nuclear fraction of FoxO1 protein [29], we tested whether JNK phosphorylation was induced by APN treatment in RANKL-treated osteoclast-precursor cells. Western blot analysis of whole protein extracts showed that JNK phosphorylation levels were dramatically enhanced by APN treatment in RAW264.7 undergoing RANKL-induced osteoclastogenesis (Figure 3B, *P*<0.05). We then evaluated if these results were consistent with the notion that APN was promoting the activation of FoxO1 upon JNK-phosphorylation. To that end, nuclear extracts from RAW264.7 undergoing RANKL-induced osteoclastogenesis in the presence and absence of APN were subjected to western blot analysis. In these experiments we found that APN treatment significantly increased FoxO1 nuclear protein levels (Figure 3C, *P*<0.05). Cytoplasmic FOXO1 levels were also increased approximately 5-fold in the presence of APN and RANKL (data not shown). However, when cells undergoing osteoclastogenesis were pretreated with the JNK inhibitor SP600125, nuclear FoxO1 levels were significantly decreased (Figure 3C, *P*<0.05). These results supported our hypothesis that APN could activate FoxO1 in a JNK-dependent manner in our *in vitro* model of osteoclastogenesis.

### FoxO1 Over-Expression Inhibits Osteoclastogenesis

**Figure 4:**
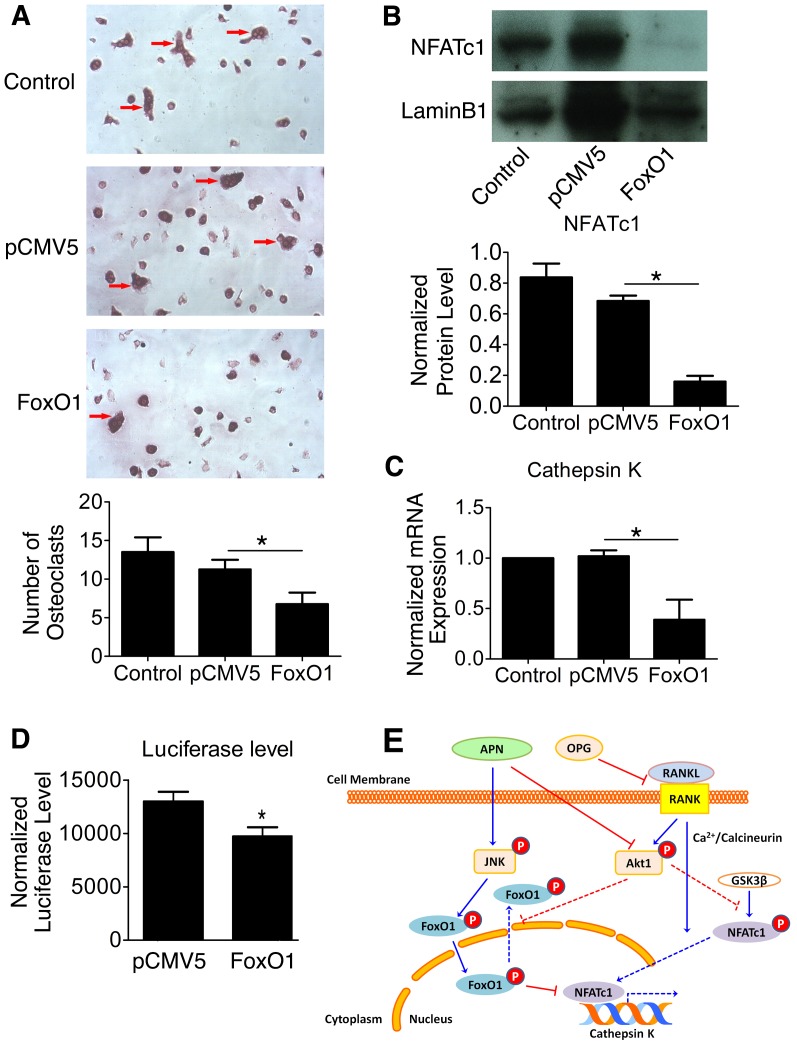
FoxO1 Over-Expression Inhibits RANKL-Induced Osteoclastogenesis by Down Regulating NFATc1. (A) BMMs isolated from WT mice were transfected with or without pCMV5 or Flag-FoxO1, and osteoclastogenesis was induced with 10 ng/ml M-CSF and 50 ng/ml RANKL for 7 days. TRAP staining was performed and the number of osteoclasts was manually counted in four separate fields (magnification, ×200; red arrows = osteoclasts). (B) Western blot for NFATc1 nuclear protein extracted from RAW264.7 cells, which were transfected with or without pCMV5 or Flag-FoxO1. Lamin B1 was detected as the loading control. Data are shown as mean ± SD of three independent experiments. *P<0.05. (C) qRT-PCR of cathepsin K mRNA levels in RAW264.7 cells transfected with or without pCMV5 or Flag-FoxO1 during osteoclastogenesis. The mRNA level was normalized to GAPDH. (D) Luciferase assay determined luciferase levels in RAW264.7 cells co-transfected with Flag-FoxO1 or pCMV5 (control) and pGL3-CtspK-luciferase reporter vector. (E) APN inhibition of RANKL-induced osteoclastogenesis through activation of FoxO1 and inactivation of NFATc1. APN promotes FoxO1 activation directly in a JNK-dependent manner and indirectly by inhibiting AKT phosphorylation [19] (data not shown). In the absence of APN, NFATc1, the master regulator of osteoclastogenesis [33], [34], is activated by RANKL in a Ca^2+^/calcineurin-dependent manner. In the presence of APN, NFATc1 nuclear translocation is inhibited indirectly by APN-mediated inhibition of AKT [19] and by a FoxO1-mediated mechanism. Inhibitory signaling by APN is depicted by red lines and stimulatory signaling is represented by blue lines. Dashed lines are used to represent signaling events that are diminished in osteoclast-precursor cells undergoing RANKL-induced differentiation in the presence of APN.

To further investigate whether APN-induced FoxO1 activation was partly responsible for the inhibitory effects of APN in osteoclastogenesis, we over-expressed FoxO1 in BMM osteoclast precursor cells undergoing osteoclastogenesis. We then performed TRAP staining to evaluate possible differences in osteoclast formation by comparing untransfected cells, cells transfected with pCMV5 control plasmid or those transfected with Flag-FoxO1 (Figure 4A). Our experiments showed that over-expression of FoxO1 under osteoclastogenic conditions led to the formation of fewer osteoclasts than in untransfected cells or those transfected with the control plasmid (Figure 4A, *P*<0.05).

We then tested whether FoxO1 activation could mediate its inhibitory effects on osteoclastogenesis by altering the activation of NFATc1, the master regulator of osteoclastogenesis. Upon binding of RANKL to RANK, NFATc1 translocates from the cytoplasm to the nucleus and induces osteoclastogenesis by increasing expression of several genes including cathepsin K. Cathepsin K is a cysteine protease expressed predominantly in osteoclasts that is required for their bone resorptive activity. We performed western blot analyses with nuclear extracts isolated from RANKL-treated RAW264.7 cells that were transiently transfected with FoxO1. Our results showed that FoxO1-overexpressing cells undergoing osteoclastogenesis exhibited a dramatic decrease in NFATc1 nuclear expression as compared to those transfected with the control plasmid (Figure 4B, *P*<0.05).

Since cathepsin K expression is normally upregulated by NFATc1 transcription factor during osteoclastogenesis, we investigated whether its expression could be altered by FoxO1 over-expression. Our results indicated cathepsin K mRNA expression was significantly reduced in FoxO1-overexpressing cells undergoing osteoclastic differentiation by RANKL (Figure 4C, *P*<0.05). To investigate whether FoxO1 could mediate those effects by acting on the cathepsin K promoter directly, Flag-FoxO1 and pGL3-CtspK-luciferase reporter vector were co-transfected in RAW264.7 cells undergoing osteoclastogenesis. Results of the luciferase assays showed that cells over-expressing FoxO1 had a lower promoter activity than cells co-transfected with the control plasmid and the pGL3-CtspK-luciferase reporter vector (Figure 4D, *P*>0.05).

Taken together, these results suggest that APN may inhibit RANKL-induced osteoclastogenesis by increasing FoxO1 nuclear protein level and by reducing NFATc1 nuclear localization and activation (Figure 4E).

## Discussion

Adiponectin is an adipokine that exhibits insulin-sensitizing effects and potent anti-inflammatory properties [30]. Circulating levels of APN are reduced in obesity and type 2 diabetes [8], [9], whereas improvement in insulin sensitivity upon treatment with thiazolidinediones correlated with increased APN levels [12], [13]. Similarly serum APN levels were lower in patients with periodontitis than in those without the disease [10], [11] and periodontal therapeutic intervention significantly led to increased serum APN levels [14]. In this study, we first compared the periodontal destruction between WT and APN^−/−^ mice, and found that periodontitis induction led to more osteoclasts and higher infiltration of inflammatory cells in APN^−/−^ mice. These results suggested that APN deficient mice were more susceptible to periodontal disease than WT mice, although alveolar bone loss was not significantly different between both groups 10 days after periodontitis induction. Whether this is resulting from the low sensitivity of the method used to measure alveolar bone loss or from compensating effects by other circulating factors needs to be further investigated.

**Figure 5:**
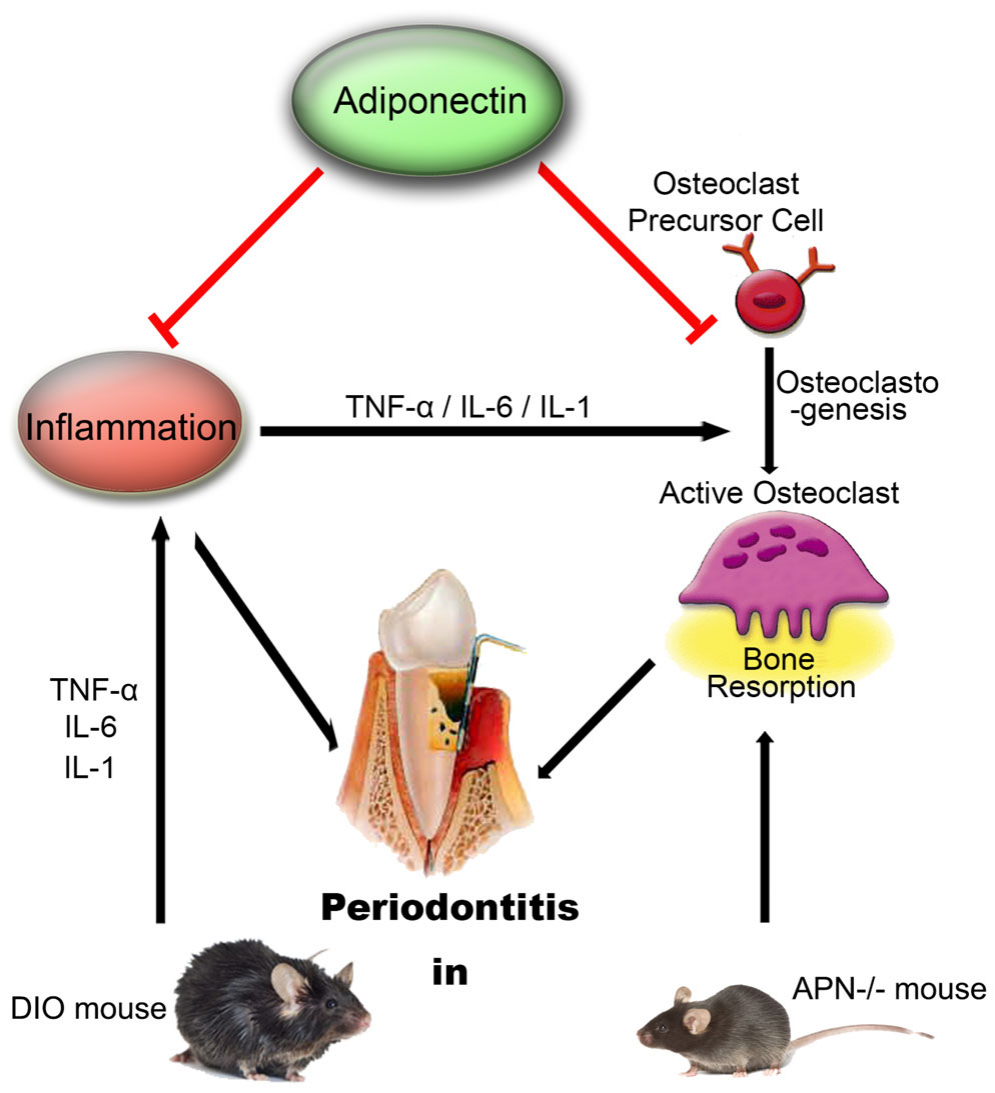
Schematic Diagram of APN Therapeutic Potential in Treating T2DM-Associated Periodontitis. We established experimental periodontitis in APN^−/−^ and DIO mice and treated them with systemic APN infusion. Our results support the notion that systemic administration with APN may constitute a therapeutic strategy to ameliorate T2DM-associated periodontitis due to dual roles of APN at inhibiting osteoclastic activity and hence bone resorption, and at attenuating inflammation.

To further determine the therapeutic potential of APN for treating T2D-associated periodontitis, we induced periodontitis in DIO mice, a model of T2D and obesity with elevated blood glucose, impaired glucose tolerance and WAT-associated chronic inflammation. Even in the context of chronic inflammation, systemic APN infusion could decrease alveolar bone loss, osteoclast number and infiltration of inflammatory cells in DIO mice induced with periodontitis. Our *in vivo* results support the notion that APN is potentially beneficial to treat T2D-associated periodontitis based on APN dual roles in inhibiting osteoclastogenic activity and attenuating local inflammation (Figure 5).

In agreement with the ability of APN to inhibit local chronic inflammation in our experimental periodontitis DIO model, we found gene expression levels of proinflammatory cytokines including TNF-α, IL-1, and IL-6 were significantly reduced in WAT tissues. WAT mediated chronic inflammation in diabetics not only contributes to more severe periodontitis [31], but also is implicated in determining insulin resistance through the production of pro-inflammatory cytokines [2], [28]. Thus, pro-inflammatory cytokines including TNF-α have been described to decrease circulatory levels of APN and promote chronic inflammation and insulin resistance. Therefore our results point out at beneficial effects of APN not only by inhibiting osteoclastogenesis and alveolar bone loss, but also by reducing chronic inflammation and hyperglycemia in DIO mice induced with periodontitis (Figure 5).

Another important finding of our study is that APN inhibited osteoclastogenesis by directly signaling through FoxO1 in osteoclast-precursor cells undergoing differentiation. FoxO1 is a transcriptional regulator of energy homeostasis [28] that controls glucose metabolism through osteoblasts partly by regulating the activity of osteocalcin. In addition, insulin signaling targets osteoblasts and inactivates FoxO1 in a PI3K/AKT dependent manner [20], [28]. A more recent study found that over-expression of FoxO family factors led to the formation of fewer mature osteoclasts [32]. This latter work was consistent with our results, showing that FoxO1 over-expression in pre-osteoclasts significantly decreased the formation of mature osteoclasts. In addition, APN increased FoxO1 gene expression in RANKL-treated RAW264.7 cells and promoted FoxO1 activation while FoxO1 over-expression decreased NFATc1 nuclear localization. NFATc1 is a master regulator of osteoclastogenesis [33], [34], and its nuclear translocation and activation up-regulates gene expression of osteoclastogenic markers hence promoting osteoclast formation. It has been reported that APN not only inhibited NFATc1 induction via AMPK signaling [6], but also by promoting its nuclear exclusion via inhibition of the Akt signaling pathway [19]. Taken together our results indicate that APN activates FoxO1 in osteoclast-precursor cells undergoing differentiation, and that FoxO1 activation inhibit RANKL-induced osteoclastogenesis by restricting NFATc1 transcription functions during osteoclastogenesis.

Whereas APN has been reported to directly inhibit bone formation by decreasing the nuclear levels of FoxO1 in a PI3K/AKT-dependent manner in osteoblasts [22], we have found that APN inhibits RANKL-induced osteoclastogenesis by increasing the nuclear levels of FoxO1 in osteoclast-precursor cells. These results are in agreement with our previous findings that APN inhibits AKT activation in RANKL-induced osteoclasts [19]. In fact, inhibition of AKT by APN has been shown to promote activation of FoxO1 indirectly, via suppressing its AKT-induced nuclear exclusion [29]. As part of our study, we further showed that APN can also activate FoxO1 in a JNK-dependent manner in osteoclast-precursor cells and that JNK-inhibition decreased APN-induced activation of FoxO1 in osteoclast-precursor cells undergoing differentiation. FoxO1 belongs to the O class of the Forkhead superfamily and is regulated by Akt and JNK signal pathways. The present study and our published work [19] demonstrated that APN activated JNK signaling and suppressed Akt signaling [19], all of which can activate FoxO1. Several reports have previously shown APN can activate JNK in a variety of cell types and tissues to regulate a variety of functions including proliferation and apoptosis. It has been reported that APN regulated the proliferation and apoptosis of C2C12 myocytes, prostate cancer and hepatocellular carcinoma cell lines via activation of JNK [35], [36]. In addition osteoblasts [37], chondrocytes [38], endothelial cells [39] and macrophages were also regulated by adiponectin through activation of JNK [40], [41] Therefore JNK and Akt signaling pathways may participate in APN-induced FoxO1 activation in osteoclasts. Hence, the different responses to APN in osteoblasts and osteoclasts may be dependent on the signaling pathway that APN activates. Further studies will be needed to characterize the underlying mechanisms involved.

In the present study, we found that APN increases FoxO1 expression in RANKL-treated RAW264.7 cells, but not in the absence of RANKL (data not shown). Although we did not evaluate whether APN by itself activates JNK in the present experiment conditions, other studies reported that APN could regulate the functions of RAW264.7 macrophages via activation of JNK [40], [41]. The synergistic effect of APN and RANKL to induce FoxO1 expresssion and JNK activation may be attributed to oxidative stress. Several reports directly suggest that oxidative stress is responsible for RANKL-induced osteoclastogenesis and bone resorption [42]–[45] In the presence of oxidative stress, JNK was phosphorylated, and further regulating FoxO1 activity, which is suggested to antagonize oxidative stress [46] Hence APN may inhibit osteoclastogenesis through modulating oxidative stress and further activating JNK and FoxO1 [47].

Although our results point out at APN inhibiting osteoclastogenesis and hence bone resorption *in vitro* and *in vivo*, they do not exclude other mechanisms that can also contribute at preserving alveolar bone integrity. In fact, signaling mechanisms through which APN affects bone metabolism are just beginning to be elucidated. Indeed, the bone phenotype of APN^−/−^ mice was shown to be normal under physiological conditions [17], [18]. However, if the effects of long term adaptation and compensation are excluded and the possible effects of mechanical loading on bone metabolism are eliminated through bone explantation assays in APN^−/−^ mice, APN inhibits osteoclastogenesis and promotes osteogenic differentiation [19]. Furthermore, sustained release of APN was also shown to improve peri-implant osteogenesis in ovariectomized rabbits by suppressing osteoclastic activity both *in vivo* and *in vitro* [21]. Importantly, our results do not exclude the possibility that APN not only inhibits osteoclastogenesis, but also promotes alveolar bone formation. In agreement with this view, it has been recently shown that APN signals in neurons of the locus coeruleus, through FoxO1 to decrease the sympathetic tone thereby increasing bone mass by decreasing energy expenditure [22]. This indirect effect on increasing bone mass has been shown to mask the opposing local effects of APN on osteoblasts at decreasing bone mass and circulating osteocalcin levels [22]. Taken together, the direct and indirect role of FoxO1 to inhibit osteoclastogenesis appears to play a dominant role over the opposite effects of APN in osteoclastogenesis through osteoblast regulation of OPG and RANKL levels. In addition, some of the protective effects of APN on alveolar bone loss found in our study may be resulting from APN inhibiting pro-inflammatory cytokines and/or from APN central effects that may increase alveolar bone mass indirectly.

In conclusion, our study supports the notion that APN may constitute a potential intervention therapy to treat T2D-associated periodontitis due to its dual inhibitory effects in bone resorption and inflammation. It also proposes that APN protection from alveolar bone loss associated with periodontitis may occur partly through a JNK-FoxO1 pathway in osteoclasts.
